# Oral carcinoma after hematopoietic stem cell transplantation – a new classification based on a literature review over 30 years

**DOI:** 10.1186/1758-3284-1-29

**Published:** 2009-07-22

**Authors:** Astrid LD Kruse, Klaus W Grätz

**Affiliations:** 1 Department of Craniomaxillofacial and Oral Surgery, University of Zurich, Zurich, Switzerland

## Abstract

**Background:**

Patients undergoing hematopoietic stem cell transplantation (HSCT) have a higher risk of developing secondary solid tumors, in particular squamous cell carcinoma, because of several risk factors, including full-body irradiation (TBI), chemotherapy, and chronic graft versus host disease (GVHD). Based on the review presented here, a classification of oral changes is suggested in order to provide a tool to detect high-risk patients.

**Methods and Results:**

The literature over the last 30 years was reviewed for development of malignoma of the oral cavity after HSCT. Overall, 64 cases were found. In 16 out of 30 cases, the tongue was the primary location, followed by the salivary gland (10 out of 30); 56.4% appeared in a latency time of 5 to 9 years after HSCT. In 76.6%, GVHD was noticed before the occurrence of oral malignancy. Premalignant changes of the oral mucosa were mucositis, xerostomia, and lichenoid changes, developing into erosive form.

**Conclusion:**

All physicians involved in the treatment of post-HSCT patients should be aware of the increased risk, even after 5 years from the development of oral malignancy, in particular when oral graft versus host changes are visible. In order to develop evidence based management, screening and offer adequate therapy as early as possible in this patient group, multicenter studies, involving oncologists and head and neck surgeons, should be established.

## Introduction

**Figure 1:**
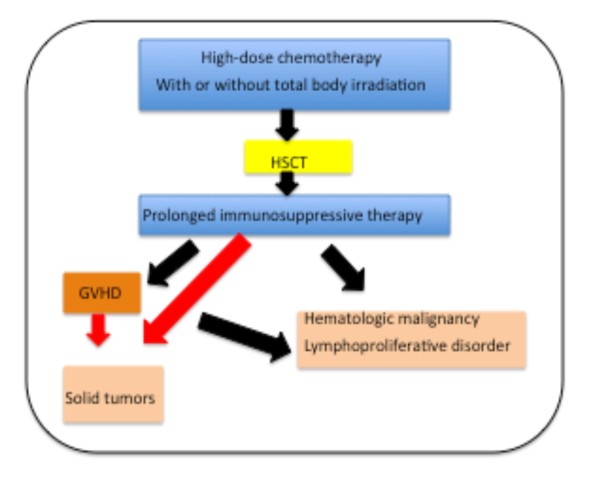
**Development of oral SCC after HSCT or GVHD**.

Allogenic hematopoietic stem cell transplantation (HSCT) has been increasingly used for therapy in recent years, leading to improvements in the survival rate. Despite this improvement, HSCT can also lead to long-term complications like the development of malignant neoplasm, which can be divided into three main subgroups: hematologic malignancy, lymphoproliferative disorder, and solid tumor. The first two are more common, developing after the transplantation, while the last one seems to be rare, and it occurs after both HSCT and GVHD [1] (Fig. 1).

In GVHD, a common complication in patients who have been treated with HSCT, immunocompetent donor T cells attack the genetically disparate host cells [2]. Billingham [3] described in 1966 three conditions for the development of GVHD. First, the graft must contain immunologically competent cells. Second, the recipient must reveal expression of tissue antigens that are not present in the donor; and finally, the recipient must be incapable of rejecting the transplanted cells. Nowadays, it is well accepted that these immunologically competent cells are donor T cells that react against histocompatibility antigens. Therefore, the degree of HLA mismatch is one of the risk factors for the development of GVHD in addition to sex mismatch, patient age, donor parity, choice of graft source, pre-infusion graft modulations, and T cells depletion [4]. In the pathogenesis process, tissue damage occurs first, caused by radiation/chemotherapy; second, donor T cells recognize host antigens from the damaged tissue as foreign, and finally, they are activated [5].

By definition, acute GVHD develops within 100 days, whereas the chronic form develops after 100 days [6]. The latter form is described in up to 40% of long-term survivors after HSCT [7].

The secondary solid tumors after GVHD include squamous cell carcinoma (SCC), melanoma, glioblastoma, and sarcoma [8]. Carcinomas have been reported in the lungs, liver, skin, parotid gland, and oral mucosa.

In general, risk factors for development of oral squamous cell carcinoma in patients without HSCT are regular tobacco and alcohol consumption, HPV, insufficient mouth hygiene, immune deficiency, and the presence of lichen planus as a premalignant lesion. The last is also described as an oral manifestation of chronic GVH.

Because of the rare appearance of oral carcinoma in patients who have undergone HSCT, the purpose of the present paper is to review published cases of this entity from the last 30 years, with and without GVHD, in order to evaluate potential risk and prognosis factors for the development of oral carcinomas. Furthermore, until now no classification has been available for changes in the oral mucosa after HSCT. Therefore, a clinical classification will be presented with emphasis on these risk factors.

## Materials and methods

For this literature review, a literature search was done using the Internet-based PubMed (National Library of Medicine and National Institute of Health, USA), limiting the search to reports of clinical trials, review articles, meta-analyses, and case reports published in English and German medical and dental journals from 1978–2008. Patients with Fanconi's anemia (FA) were excluded because they have an inborn susceptibility to cancer. The hazard rate for cancer in FA patients was reported to be 2% per year by the age of 24 years with a cumulative incidence of 29% by the age of 48 years (Alter et al. 2003).

## Results

**Figure 2:**
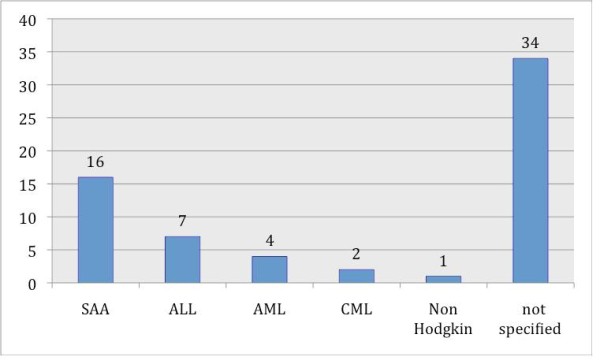
**Distribution of primary diagnosis**.

**Figure 3:**
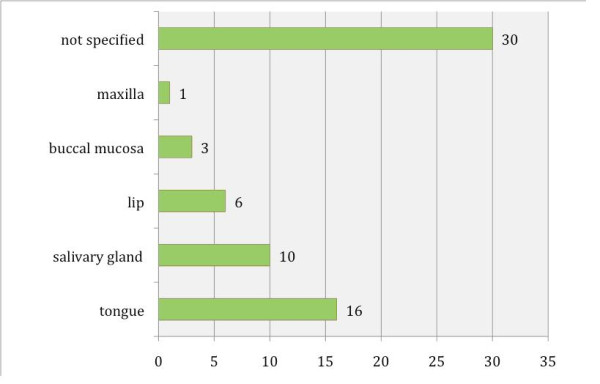
**Distribution of localization**.

**Figure 4:**
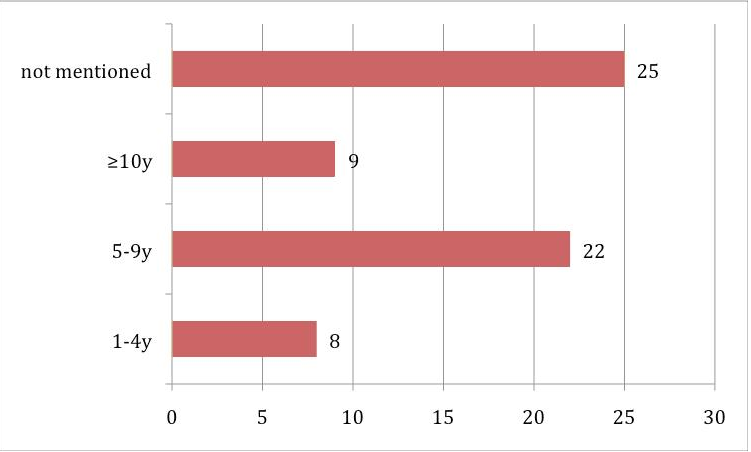
**Latency time (years) between HSCT and oral malignancy**.

**Figure 5:**
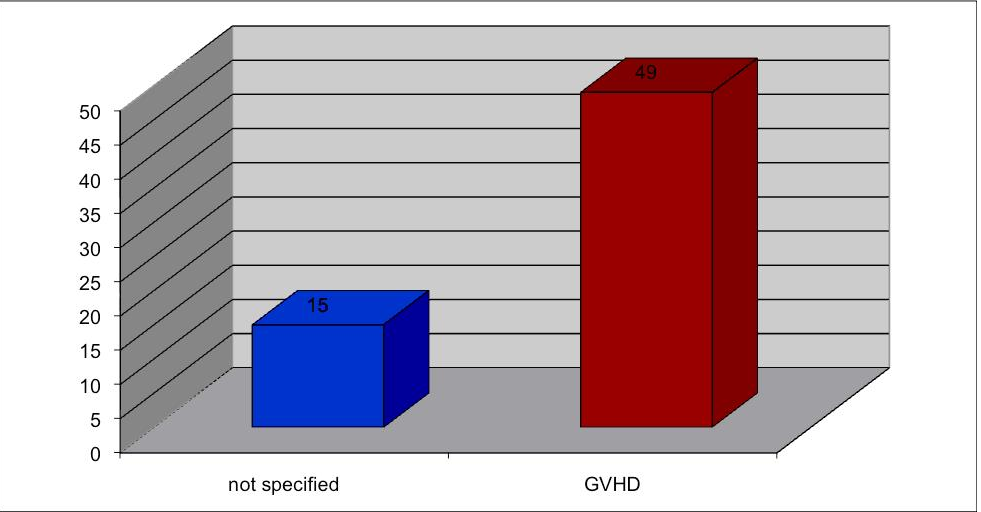
**Distribution of patients with previous GVHD**.

**Figure 6:**
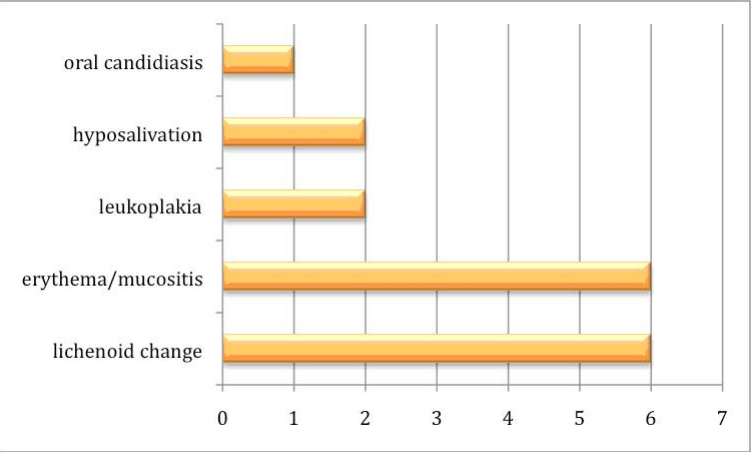
**Distribution of premalignant oral conditions**.

Between 1978 and 2008, 5 case reports and 12 studies involving oral carcinoma in combination with HSCT were published, including 64 cases in all (Table S1; Additional File 1) [9-24]. In 40 cases the gender distribution was not specified; the male-to-female distribution was 19:5 in all other cases. In 16 of 30 specified original diseases, SAA (severe aplastic anemia) was predominant (Fig. 2). In 30 patients the localization of the oral malignant neoplasm was not specified; in all other cases the tongue (16 out of 30) was the most common location, followed by the submandibular gland (8 out of 30) (Fig. 3). Concerning the distribution of latency time between HSCT and the appearance of oral malignancy, 39 cases were specified; the main group was between 5 and 9 years (56.4%), followed by the group for 10 years or more (23.1%) (Fig. 4). In 76.6% GVHD was observed before the occurrence of oral malignancy (Fig. 5). Over the 5 case reports, 9 cases were presented with oral GVHD; the oral changes are presented in Figure 6.

**Figure 7:**
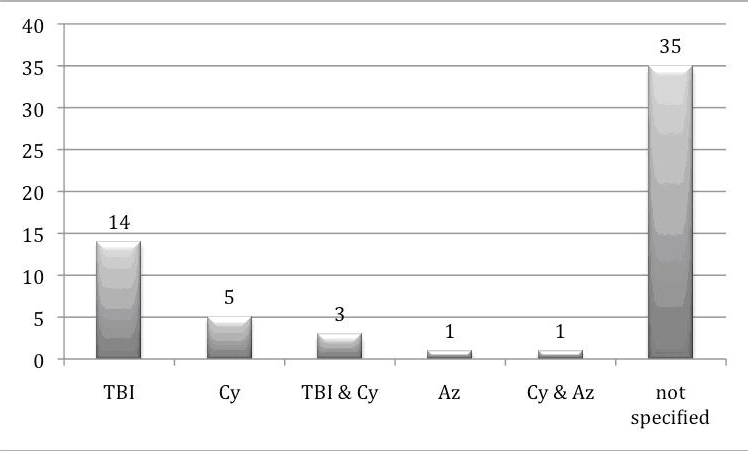
**Distritubtion of pretreatment**.

The pretreatment was specified in only 29 cases (Fig. 7); total body irradiation (TBI) was performed in 17 cases.

## Discussion

The incidence of secondary neoplasm for cancer of the oral cavity, esophagus, or thyroid gland in patients with previous HSCT is 4 to 7 times that of the general population [16,24,25]. Several risk factors for occurrence of solid tumors after HSCT have been discussed in the literature, including total-body radiation [26-28], chemotherapy [15], male gender [11], virus [8,29], young age [16], chronic GVHD [24,28], and prior immunosuppressive therapy [11].

The present gender distribution of 19:5 supports the observation by Gluckman [11] and Bhatia [28] that males have a higher risk than females for development of secondary oral malignancy, though males are only slightly more likely than females to receive HSCT (62% to 56%) [11,15,28].

Even though some authors have proposed total-body irradiation as an independent risk factor for solid tumors [25-27], others [15] do not support this. In the present study for oral malignancy, in only 29 cases was the pretreatment specified, and of those, 19 had total-body radiation (Fig. 7). In addition, Hojo et al. (1999) [30] proposed that cyclosporine can promote cancer progression by a direct cellular effect that is independent of its effect on the host's immune cells. This statement was supported by Kolb et al. [15], who described immunosuppressive treatment, in particular cyclosporine and azathioprine, as the most significant risk factor for secondary malignant neoplasm. In the current study data from only 10 patients, information on cyclosporine and/or azathiorprine are available; thus a statement concerning their influence on the development of oral cancer and comparison to patients under immunosuppression because of organ transplants is not possible due to lack of data.

Kolb (1999) [15] described a risk for a malignant neoplasm of 3.5% at 10 years after HSCT and 11.5% at 15 years, with the overall incidence of malignant tumors being about 5-fold greater than that in an age-and-sex-matched population and more than 10-fold greater for cancer of the oral cavity, esophagus, or thyroid gland. In the current overview, the latency time between HSCT and occurrence of oral malignancy in the group between 5 and 9 years was increased. Instances in the group of more than 10 years would likely be higher, because GVHD contributes to significant post-HCT morbidity and mortality. In the present study 47 patients had already revealed the occurrence of the oral cancer GVHD. In most studies the differentiation concerning the specification of the oral form of GVHD is missing. But in the 9 case reports in all cases, an oral involvement of GVHD was described. This oral form is characterized pathohistologically by mucosal hyperkeratosis, erythema, atrophy, inflammation, pseudomembranous ulceration [5], fibrosis, and salivary gland disorder [31]. Probably one reason for the rare data in the studies could be the difficulty of the differential diagnosis of oral GVHD like neutropenia-associated mucositis, recrudescent HSV, or cytomegalovirus stomatitis [32].

Controversy still surrounds the etiology of oral SSC in-patients with GVHD. Lichenoid changes of the oral mucosa in combination with oral GVHD were present in 6 of the 9 single case reports. Because lichen has a malignant potential, the malignant transformation in GVHD is probably based on the development of oral lichen. Under both conditions (without previous HSCT and after HSCT), basal cells are the prime target of destruction, and T lymphocytes become cytotoxic for basal keratinocytes [33]. For the onset of OLP, an immunological cause related to coinfection and administration of different medication is discussed [34]. Therefore, the question arises whether total-body radiation with application of immunosuppressive agencies like cyclosporine/azathioprine can lead to the development of lichen in patients after HSCT. Bradfort (1990) [35] presented 2 cases after organ transplantation and treatment with azathioprine that developed a squamous cell carcinoma, but in one case the patient had an ethanol and nicotine (100 py) consumption; thus the risk of developing an oral squamous cell carcinoma was already considerable.

Apart from the fact that the most common localization in the oral cavity was the tongue (6/35), the number of salivary gland malignoma (10/35) is striking: from those 10 cases, only 5 of the tumors have been verified, and all 5 were mucoepidermoid carcinomas. Some authors compare the clinical features of GVHD with those of other autoimmune connective tissue disorders, like progressive systemic sclerosis and Sjörgen's syndrome [36]; in both those diseases, hyposalivation is described. Nakamura [37] shows that periductal lymphocytic infiltratin is typical in Sjorgens's syndrome but not in GVHD patients, while Nicolatou-Galltis (2000) [38] describes mild periductal monocytic infiltration in GVHD patients.

The described xerostomia as a cause of oral GVHD was mentioned in 2 of the 9 single case reports. Mucoepdermoid and squamous cell carcinoma in salivary gland tumors originate from the excretory duct, but in the current literature, there has been no investigation concerning the histopathological changes in these excretory ducts as a cause of oral GVHD.

Concerning therapy for GVHD several strategies have been discussed, but because of missing data, evidence for management is still missing. The most common seems to be cyclosporine in combination with corticosteroids for regression of T cell proliferation; one of the disadvantages after long-term use can be an increased incidence of squamous cell carcinoma [39]. Second, Tacrolimus has an overall response rate of 20%. Third, Sirolimus is described as having a reduced risk of gingival overgrowth [39]. But in all single case reports, patients had previously revealed an oral manifestation of GVHD and nevertheless developed, particularly in the oral cavity, a malignant neoplasm. In the literature no data are available on how many patients with a treated oral GVHD did not develop an oral malignancy.

The main limitation of this study was that the data were from big studies summarizing all secondary tumors after HSCT, thus resulting in a lack of specific data for tumors of the oral cavity. Most of the big studies provided information independent of the tumor localization, so precise data concerning, e.g., oral manifestation of GVHD were not available.

Very few studies described the follow-up [18], but the squamous cell carcinoma of the oral cavity after HSCT seems to be very aggressive [18,23], and therefore early detection is extremely important. Therefore, based on the descriptions of intraoral premalignant conditions and the occurrence of oral GVHD in the current available studies, a classification (Table 1) is suggested in order to provide a tool to detect high-risk patients. In all cases other risk factors, such as smoking or regular alcohol use, should be avoided. Besides the systemic therapy in cases of GVHD, in cases of erythema/mucositis particular attention should be paid also to optimization of oral hygiene and mouth rinsing with cyclosporine, along with a strict follow-up. Lichenoid appearance must be controlled regularly in order not to miss erosive changes that need to be biopsied.

**Table 1:** Classification of clinical intraoral changes after hematopoietic stem cell transplantation

**Grade**	**Clinical intraoral features**
0	No involvement
1	Erythema and/or hyposalivation
2	Lichenoid appearance
3	Ulceration, tumor

## Conclusion

All physicians treating patients post-HSCT, even after 5 years or more, should be aware of the increased risk of developing oral malignancy, in particular when oral graft versus host changes are visible. Many questions concerning the etiology are still not solved; therefore, multicenter studies that involve oncologists and head and neck surgeons should be established in order to investigate the pathogenesis and also to detect as early as possible patients at high risk for oral malignancy in order to offer adequate therapy.

## Competing interests

The authors declare that they have no competing interests.

## Authors' contributions

AK carried out the analysis of literature and drafted the manuscript. KWG participated in the design of the study and the coordination. Both authors read and approved the final manuscript.

## Supplementary Material

Additional file 1 **Table S1**. Overview of all published cases of secondary oral malignoma after HSCT Ns = not specified. Click here for file
