# Supplementary material for: Oral carcinoma after hematopoietic stem cell transplantation – a new classification based on a literature review over 30 years
Source: Head Neck Oncol. 2009 Jul 22;1:29. doi: 10.1186/1758-3284-1-29 (PMC2724375; doi:10.1186/1758-3284-1-29)
Supplement: Additional file 1 — Table S1. Overview of all published cases of secondary oral malignoma after HSCT Ns = not specified. [file 1758-3284-1-29-S1.doc]

| **Author** | **Patients** | **Sex** | **Primary diagnosis** | **localisation** | **GVHD** |
| --- | --- | --- | --- | --- | --- |
| Deeg (1984)  [8] | 1 | m | ALL | buccal mucose | yes |
| Witherspoon (1989) [9] | 2 | ns  ns | ns  ns | Tongue  ns | yes  no |
| Lishner (1990) [10] | 1 | m | SAA | Lower lip | yes |
| Gluckman (1991) [11] | 4 | ns  ns  ns  ns | SAA  SAA  SAA  SAA | lip or mouth mucosa  lip or mouth mucosa  lip or mouth mucosa  parotis | yes  yes  yes  yes |
| Socie (1992) [12] | 4 | m  m  m  m | SAA  SAA SAA  SAA | oral cavity  parotis  lip  cheek | yes  no  yes  yes |
| Witherspoon (1992) [13] | 2 | m  m | SAA  SAA | tongue  maxilla | yes  yes |
| Lowsky (1994) [14] | 2 | f  f | SAA  SAA | tongue  ns | Yes  yes |
| Kolb (1995) [15] | 7 | ns  ns  ns  ns  ns  ns  ns | ns  ns  ns  ns  ns  ns  ns | ns  ns  ns  ns  ns  ns  ns | no  no  no  yes  yes  yes  yes |
| Curtis  (1997) [16] | 16 | ns  ns  ns  ns  ns  ns  ns  ns  ns  ns  ns  ns  ns  ns  ns  ns | ns  ns  ns  ns  ns  ns  ns  ns  ns  ns  ns  ns  ns  ns  ns  ns | lip  lip  tongue  tongue  tongue  tongue  tongue  tongue  salivary gland  salivary gland  salivary gland  other side oral cavity  other side oral cavity  other side oral cavity  other side oral cavity  other side oral cavity | yes  yes  yes  yes  yes  yes  yes  yes  yes  yes  yes  yes  yes  yes  yes  yes |
| Otsubo (1997) [17] | 1 | f | SAA | gingiva | yes |
| Socie (2000) [18] | 5 | m  m  m  m  f | ALL  ALL  ALL  AML  ALL | tongue  tongue  tongue  salivary gland  salivary gland | no  yes  yes  no  no |
| Bhatia (2001) [19] | 6 | ns  ns  ns  ns  ns  ns | ns  ns  ns  ns  ns  ns | salivary gland  salivary gland  salivary gland  ns  ns  ns | no  no  no  yes  yes  yes |
| Abdelsayed (2002) [20] | 2 | m  m | ALL  ALL | buccal mucosa  tongue | yes  no |
| Zhang (2002) [21] | 3 | m  m  m | CML  CML  AML | tongue  lower lip  lower lip | yes  yes  yes |
| Baker (2003) [22] | 5 | ns  ns  ns  ns  ns | ns  ns  ns  ns  ns | ns  ns  ns  ns  ns | ns  ns  ns  ns  ns |
| Szeto (2004) [23] | 2 | m  m | AML  AML | tongue  tongue | yes  yes |
| Demarosi (2005) [24] | 1 | f | Non Hodgkin | buccal mucosa | yes |

Table 2: Overview of all published cases of secondary oral malignoma after HSCT

Ns= not specified
